# Supplementary material for: LNMAT1 promotes lymphatic metastasis of bladder cancer via CCL2 dependent macrophage recruitment
Source: Nat Commun. 2018 Sep 20;9:3826. doi: 10.1038/s41467-018-06152-x (PMC6148066; doi:10.1038/s41467-018-06152-x)
Supplement: Supplementary file 1 — Supplementary information [file 41467_2018_6152_MOESM1_ESM.pdf]

Long noncoding RNA *LNMAT1* promotes lymphatic metastasis of bladder cancer via CCL2-dependent macrophage recruitment

Changhao Chen[1,2#], Wang He[1,2#], Jian Huang[1,2#], Bo Wang[1,2], Hui Li[3], Qingqing Cai[4], Feng Su[1], Junming Bi[1,2], Hongwei Liu[1,2], Bin Zhang[5], Ning Jiang[1], Guangzheng Zhong[1], Yue Zhao[6], Wen Dong[1,2], Tianxin Lin[1,2]*

[1]Department of Urology, [2]Guangdong Provincial Key Laboratory of Malignant Tumor Epigenetics and Gene Regulation, Sun Yat-sen Memorial Hospital, State Key Laboratory of Oncology in South China, Guangdong, P. R. China

[3]Department of Biochemistry and Molecular Genetics, School of Medicine, University of Virginia, Charlottesville, VA 22908, USA

[4]Department of Medical Oncology, Sun Yat-sen University Cancer Center, State Key Laboratory of Oncology in South China, Guangzhou, P. R. China

[5]Department of Hepatopancreatobiliary Surgery, Sun Yat-sen Memorial Hospital, Guangzhou, P. R. China

[6]Department of Tumor Intervention, Sun Yat-sen University First Affiliated Hospital, Guangzhou, P. R. China;

* Correspondence to:

Tianxin Lin, M.D. Ph.D. Department of Urology, Sun Yat-sen Memorial Hospital, 107th Yanjiangxi Road, Yuexiu District, Guangzhou, Guangdong province, P. R. China, Postal code: 510120, E-mail: lintx@mail.sysu.edu.cn, Tel.:+86-13724008338, work telephone number: +86-20-34070447, fax: +86-20-81332336.

[#] These authors contributed equally to this study.

Supplementary Figures

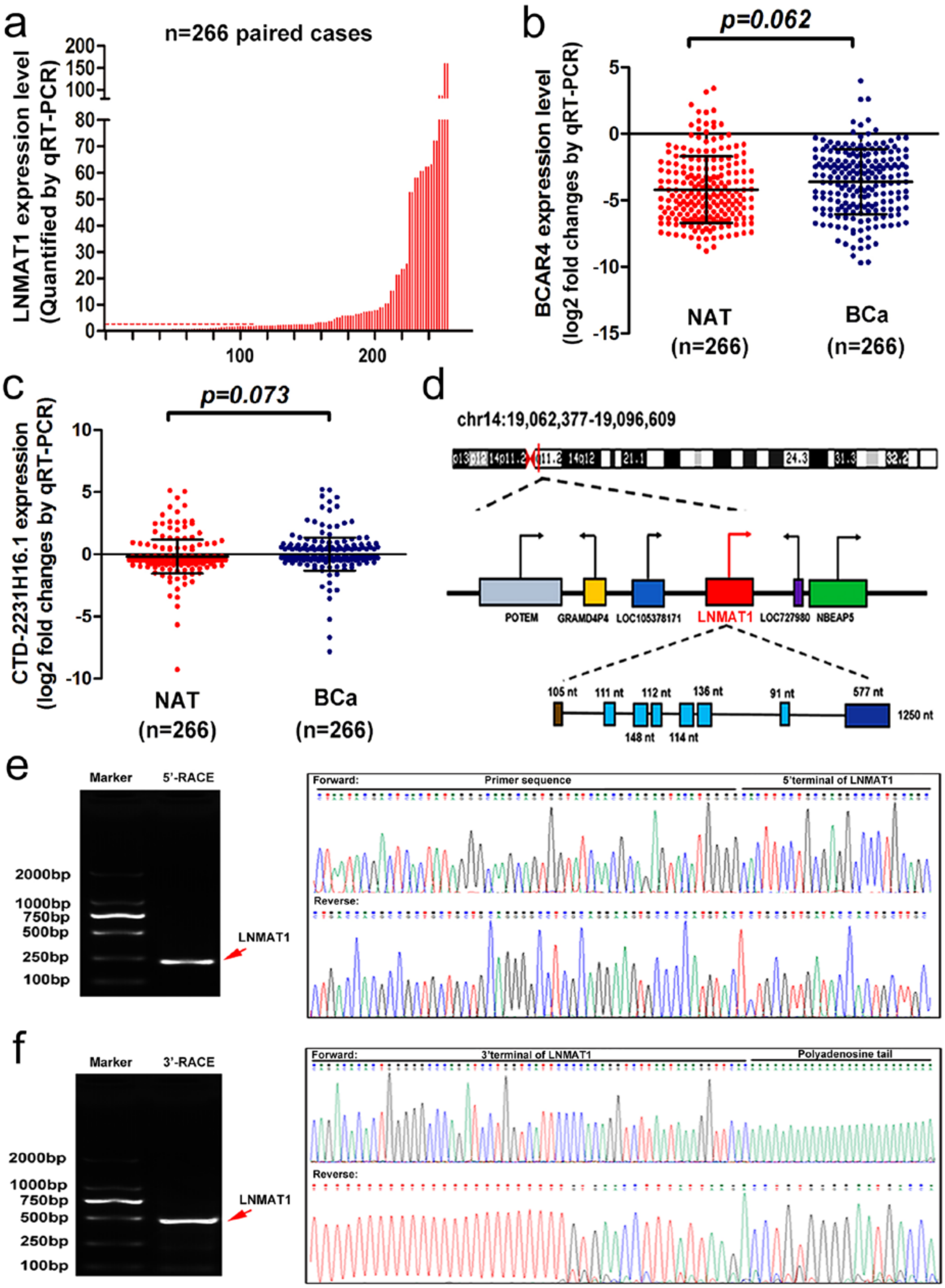

Supplementary Figure 1. | ***LNMAT1* is overexpressed in bladder cancer. (a-c)** qRT-PCR analysis of *LNMAT1*, *BCAR4* and *CTD-2231H16.1* expressions in 266 paired bladder cancer tissues and NATs. Transcript levels were normalized to GAPDH expression. **(d)** Schematic

visualization of chromosomal location of *LNMAT1*, its neighboring protein coding genes and alternative splicing isoforms of *LNMAT1* determined by RACE analysis. **(e)** Representative image of agarose gel electrophoresis and bidirectional sequencing of 5'-RACE products of *LNMAT1* showing the cap sequence and 5'-terminal sequence of *LNMAT1*. At least 10 *E. coli* clones were sequenced. **(f)** Representative image of agarose gel electrophoresis and bidirectional sequencing of 3'-RACE products of *LNMAT1* showing the tail sequence and 3'-terminal sequence of *LNMAT1*. The error bars represent standard deviations of three independent experiments. *$p < 0.05$ and **$p < 0.01$.

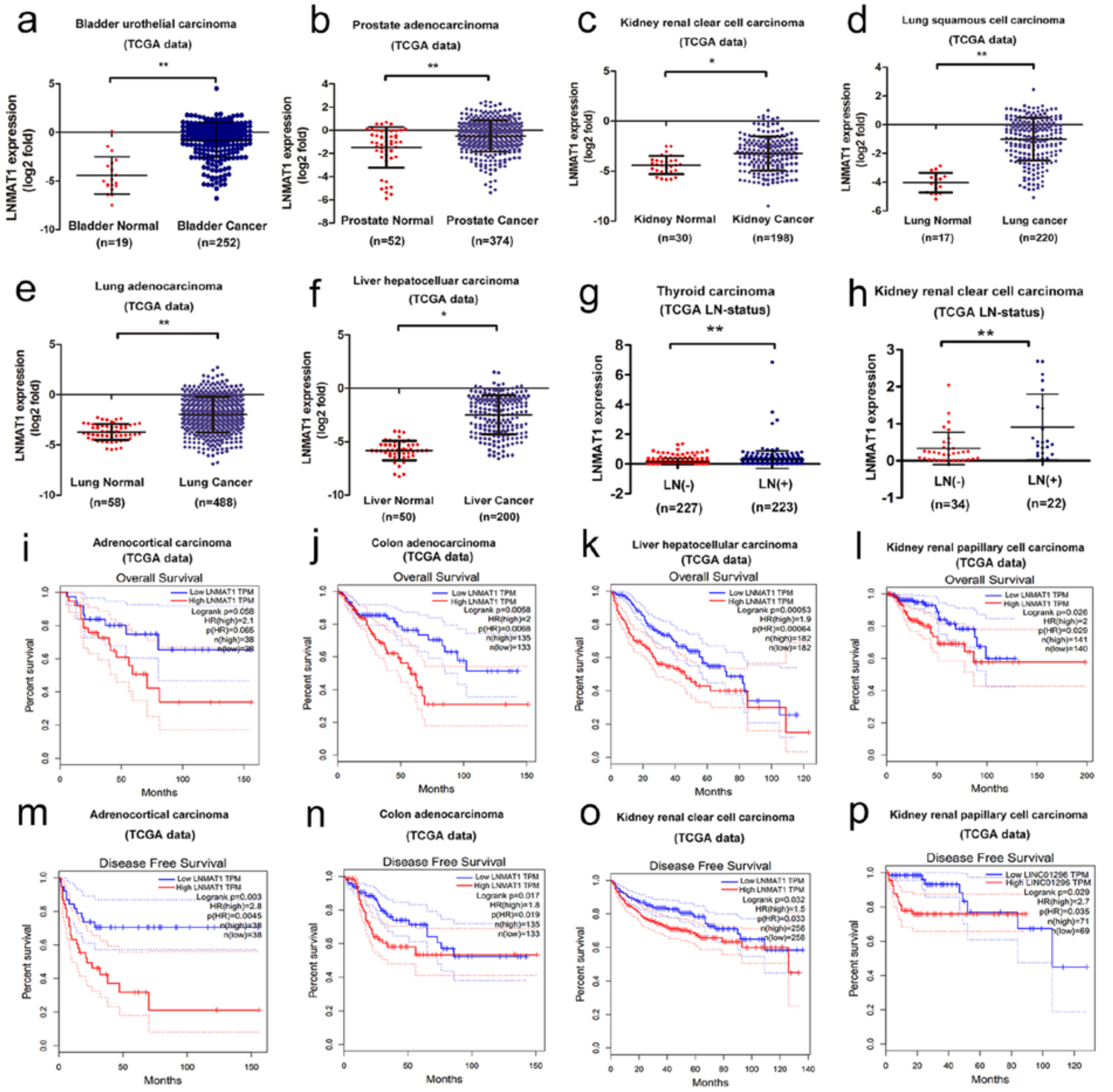

Supplementary Figure 2. | ***LNMAT1* is overexpressed in multiple types of human cancer.** **(a)** TCGA data analysis showed that *LNMAT1* is upregulated in bladder cancer tissues (n = 252) relative to normal urothelium tissues (n = 19). The nonparametric Mann-Whitney U test was used. **(b-f)** Data from the TCGA database for different types of cancers were analyzed. The nonparametric Mann-Whitney U test was used. **(g-h)** TCGA data analysis showed that *LNMAT1* is upregulated in LN metastasis tissues relative to non-LN metastasis tissues for different types of cancers. The nonparametric Mann-Whitney U test was used. **(i-p)** Kaplan-Meier survival analysis of OS and DFS different types cancer patients with expression profile of *LNMAT1*-high vs *LNMAT1*-low from TCGA databases. The median *LNMAT1* expression was used as the cutoff value. The data was obtained from GEPIA

(http://gepia.cancer-pku.cn/index.html). The log-rank (Mantel-Cox) test was used to calculate p-values. The error bars represent standard deviations of three independent experiments. *p < 0.05 and **p < 0.01.

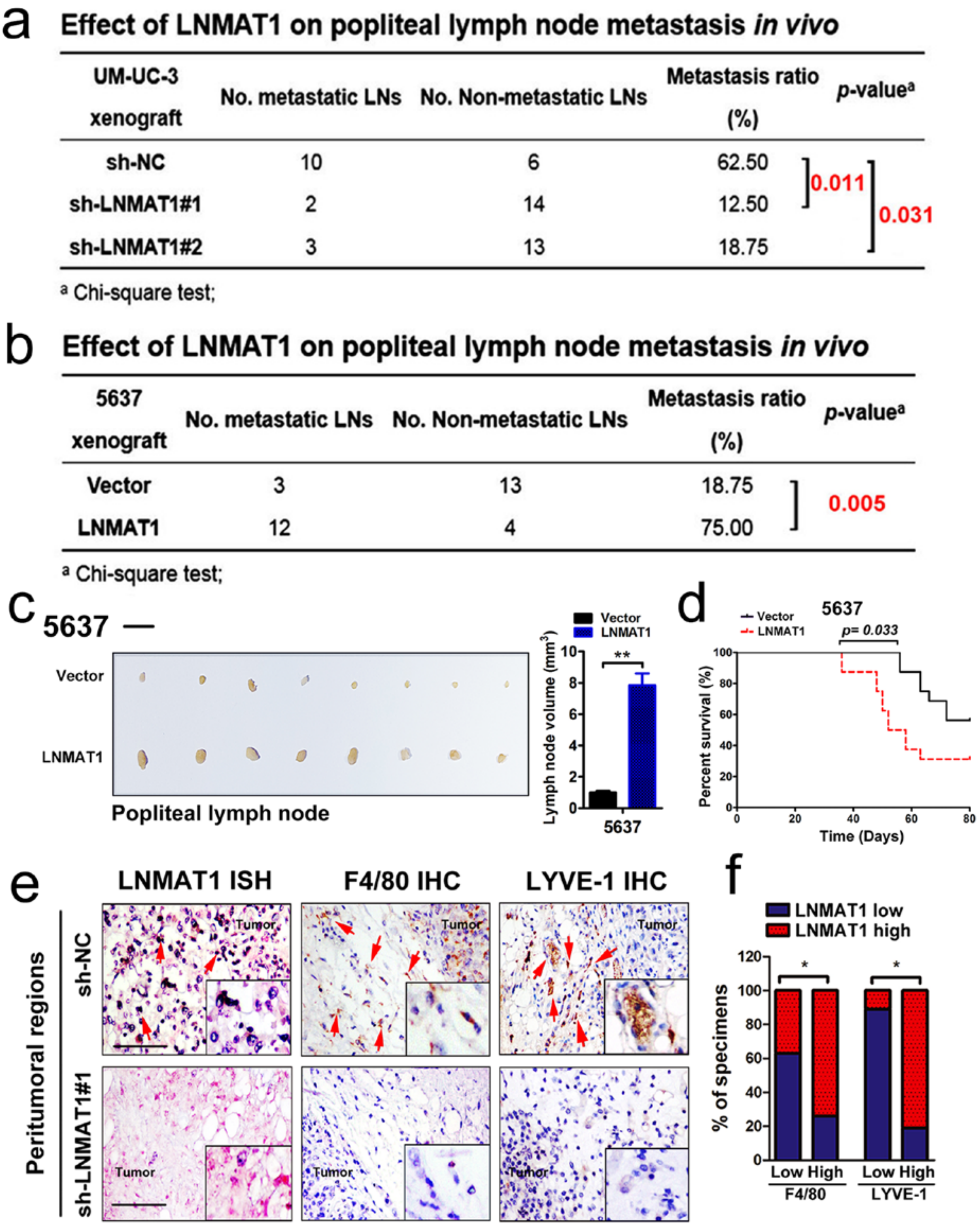

a Effect of LNMAT1 on popliteal lymph node metastasis *in vivo*

UM-UC-3 xenograft	No. metastatic LNs	No. Non-metastatic LNs	Metastasis ratio (%)	*p*-value[a]
sh-NC	10	6	62.50	
sh-LNMAT1#1	2	14	12.50	0.011 (vs sh-NC)
sh-LNMAT1#2	3	13	18.75	0.031 (vs sh-NC)

[a] Chi-square test;

b Effect of LNMAT1 on popliteal lymph node metastasis *in vivo*

5637 xenograft	No. metastatic LNs	No. Non-metastatic LNs	Metastasis ratio (%)	*p*-value[a]
Vector	3	13	18.75	0.005
LNMAT1	12	4	75.00	

[a] Chi-square test;

Supplementary Figure 3. | ***LNMAT1* overexpression promotes LN metastasis *in vivo*.**

(a,b) Ratios of the number of metastatic to total enucleated popliteal LNs for the indicated groups. Statistical significance was assessed using χ^2 test. **(c)** Representative images of enucleated popliteal LNs and histogram analysis of the LN volume in the indicated cells. Statistical significance was assessed using two-tailed t-tests. (n = 16, Scale bar: 5mm). **(d)** Kaplan-Meier (Mantal-Cox) test of the mice (n = 16) that were inoculated in the indicated cells. **(e,f)** Representative images and percentages of mice tissues with high or low levels of F4/80-positive cells and LYVE-1-positive cells in the peritumoral tissues with different *LNMAT1* expression levels. *LNMAT1* expression levels were quantified by ISH, macrophage density was quantified by IHC using anti-F4/80 antibody and microlymphatic vessel density was quantified by IHC using the anti-LYVE-1 antibody. Two representative cases are shown. Statistical significance was assessed by χ^2 test. Scale bars: 100 μm. The error bars represent standard deviations of three independent experiments. $^*p < 0.05$ and $^{**}p < 0.01$.

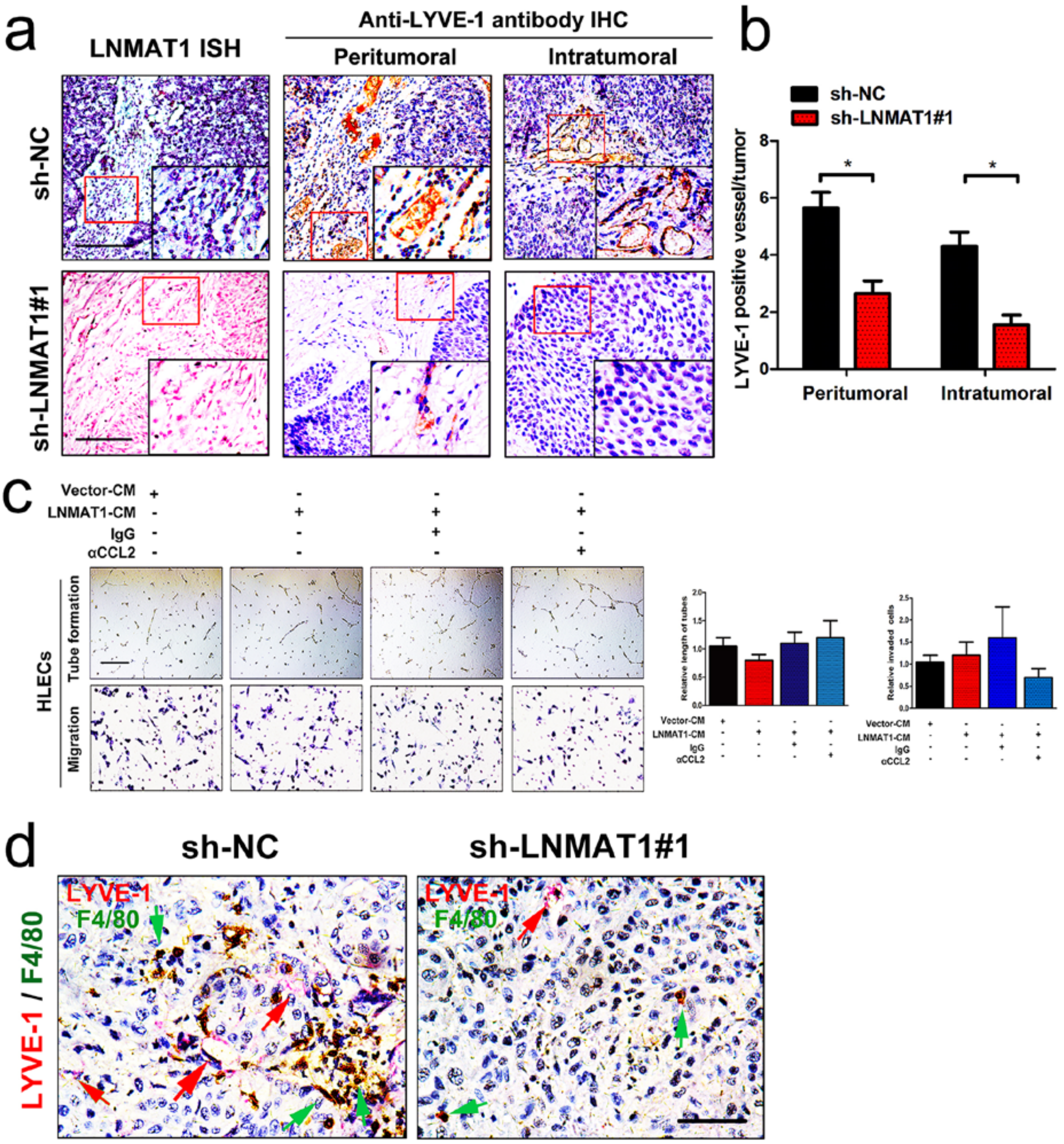

Supplementary Figure 4. | ***LNMAT1* overexpression promotes lymphangiogenesis. (a,b)** Representative images and percentages of mice tissues with high or low levels of LYVE-1-positive cells in the peritumoral or intratumoral tissues with different *LNMAT1* expression levels. *LNMAT1* expression levels were quantified by ISH. MLVD was quantified by IHC using the anti-LYVE-1 antibody. Statistical significance was assessed by χ^2 test. Scale bars: 100 µm. **(c)** Representative images and quantification of tube formation and Transwell invasion by HLECs treated with the conditioned medium collected from bladder cancer cells. Scale bars: 100 µm. Statistical significance was assessed using two-tailed t-tests. **(d)** Representative double IHC staining of LYVE-1 (red) and F4/80 (brown) in mice tissues with different *LNMAT1* expression levels. Scale bars: 50 µm. The error bars represent standard

deviations of three independent experiments. *$p < 0.05$ and **$p < 0.01$.

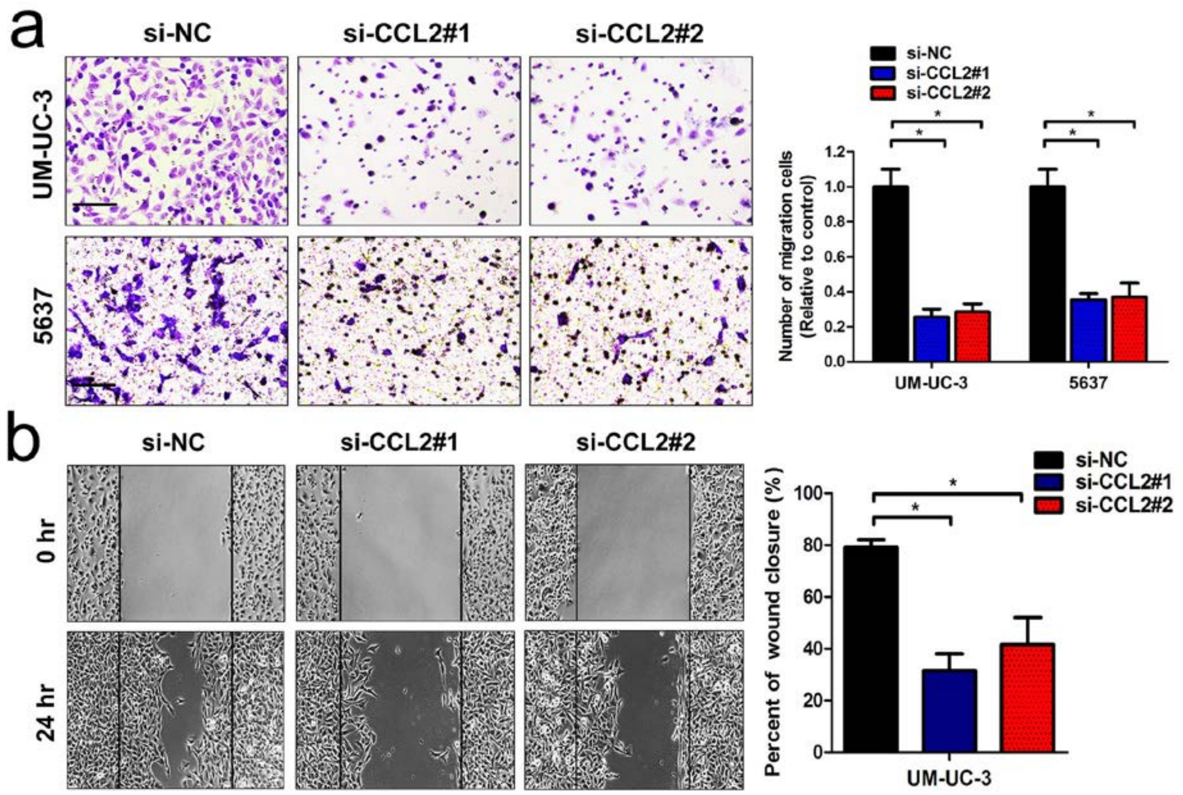

Supplementary Figure 5.｜CCL2 inhibition decreased the migratory capability. (a) Representative images of Transwell assay using UM-UC-3 and 5637 cells showing cell motility after knockdown of CCL2 (left panels) and a histogram analysis of migrated cell counts are shown (right panels). Statistical significance was assessed using one-way analyses of variance (ANOVA) followed by Dunnett's tests and two-tailed t-tests. Scale bars: 100 µm. **(b)** Representative images of wound healing assay using UM-UC-3 cells showing cell motility after knockdown of CCL2 (left panels) and a histogram analysis of cell migration distance are shown (right panels). Statistical significance was assessed using one-way analyses of variance (ANOVA) followed by Dunnett's tests and two-tailed t-tests. The error bars represent standard deviations of three independent experiments. *$p < 0.05$ and **$p < 0.01$.

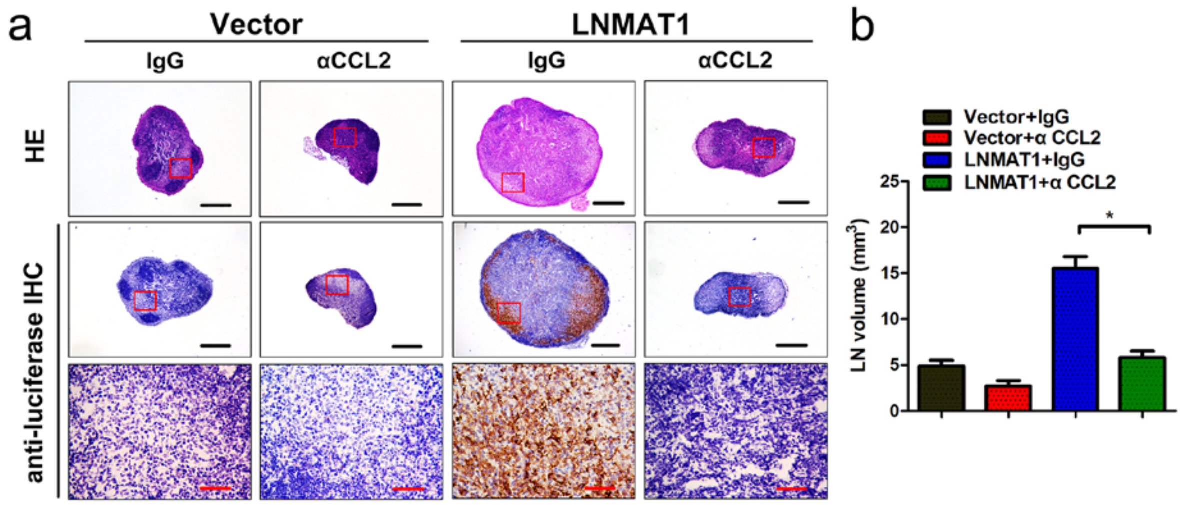

c

Effect of LNMAT1 on popliteal lymph node metastasis *in vivo*

UM-UC-3 xenograft	No. total metastasis LNs	No. total Non-metastasis LNs	Metastasis ratio (%)	*p*-value[a]
Vector	6	10	37.50	0.031
LNMAT1	13	3	81.25	
Vector+IgG	6	10	37.50	0.703
Vector+αCCL2	4	12	25.00	
LNMAT1+IgG	15	1	93.75	0.001
LNMAT1+αCCL2	3	13	18.75	

[a] Chi-square test;

d

Effect of LNMAT1 on popliteal lymph node metastasis *in vivo*

UM-UC-3 xenograft	No. total metastasis LNs	No. total Non-metastasis LNs	Metastasis ratio (%)	*p*-value[a]
Vector+sh-NC	5	11	31.25	0.683
Vector+shCCL2#1	3	13	18.75	
LNMAT1+sh-NC	13	3	81.25	0.001
LNMAT1+shCCL2#1	2	14	12.50	

[a]Chi-square test;

Supplementary Figure 6. ｜CCL2 is required for *LNMAT1*-induced LN metastasis. (a) Representative images of HE and IHC staining confirming the LN status (n=16). Scale bars: black, 500 μm; red, 100 μm. Statistical significance was assessed using two-tailed t-tests. **(b)** Volume quantification of popliteal LN metastasis after inhibition of CCL2 with neutralizing antibody. **(c,d)** Ratios of the number of metastatic to total enucleated popliteal LNs for the indicated groups. Statistical significance was assessed using χ^2 test. The error bars represent standard deviations of three independent experiments. *$p < 0.05$ and **$p < 0.01$.

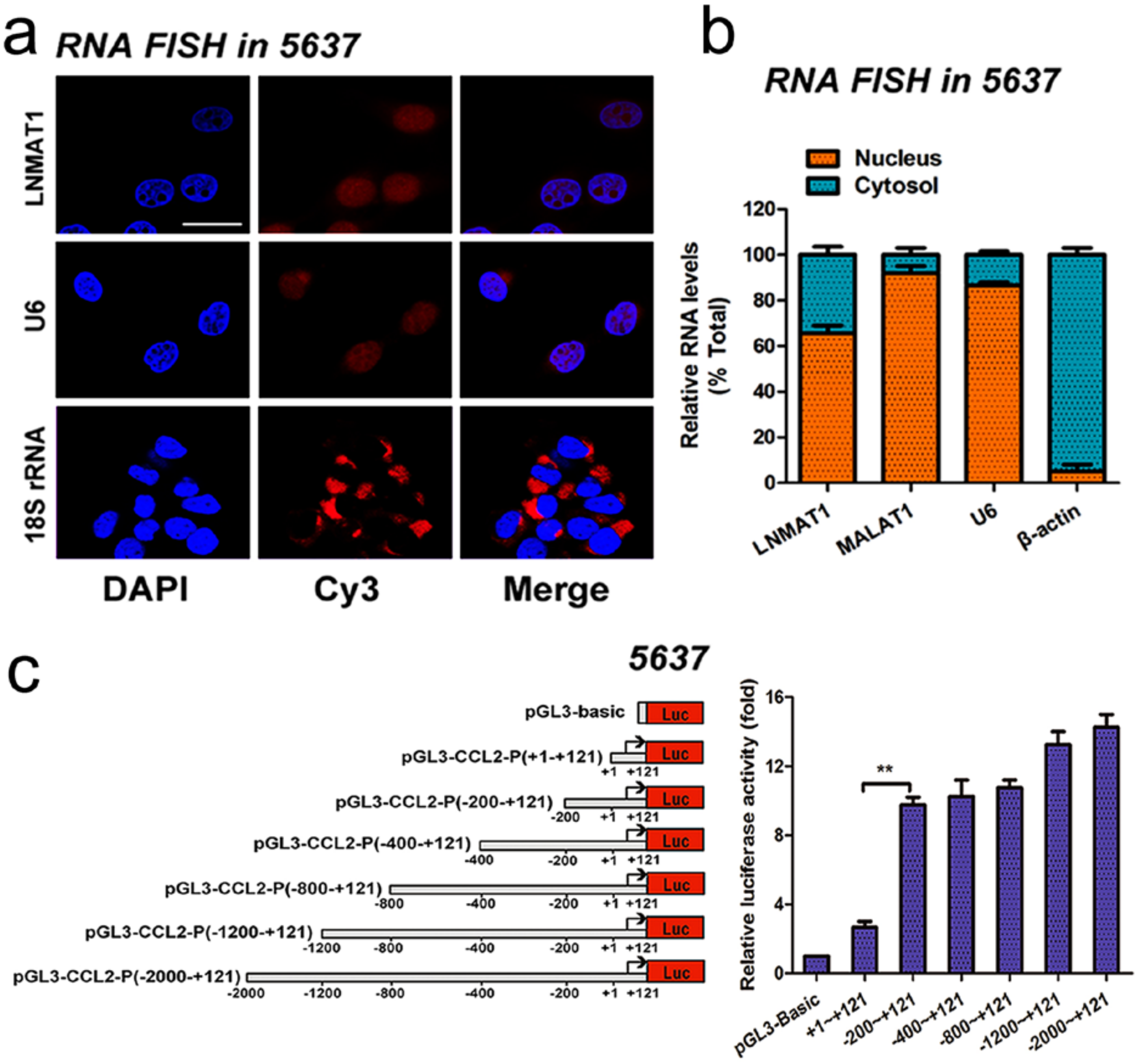

Supplementary Figure 7. | ***LNMAT1* was localized to the nucleus. (a)** FISH analysis of the subcellular distribution of *LNMAT1* in 5637 cells. Scale bar: 10 μm. **(b)** Nuclear fractionation and qRT-PCR analyses of *LNMAT1* expression in the nucleus and cytoplasm of 5637 cells. **(c)** Transcriptional activity of the CCL2 promoter was evaluated through sequential deletions in 5637 cells and by examining the CCL2 promoter linked to Renilla luciferase activity. Statistical significance was assessed using two-tailed t-tests. The error bars represent standard deviations of three independent experiments. *$p < 0.05$ and **$p < 0.01$.

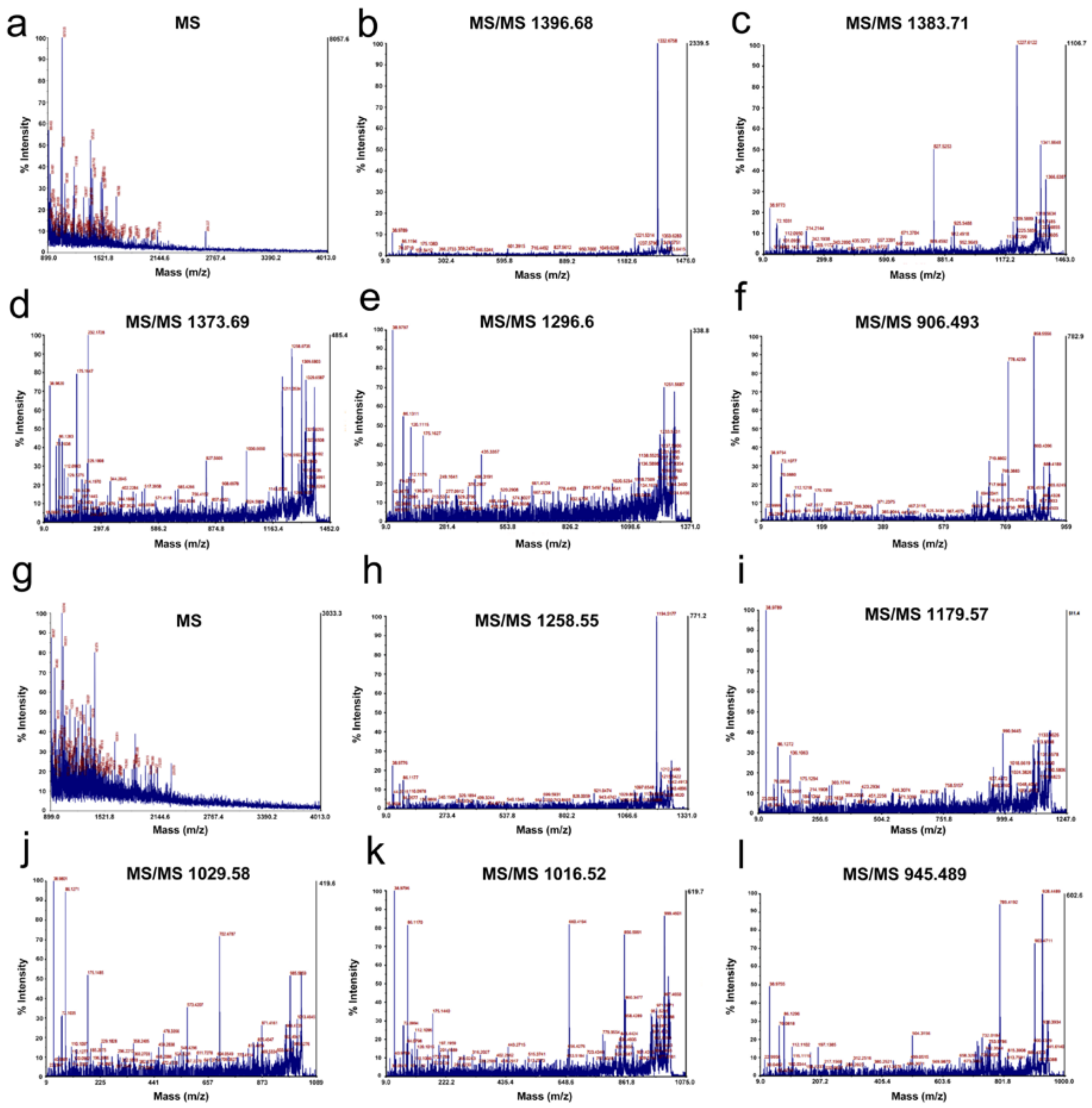

Supplementary Figure 8.｜The *LNMAT1*-interacting protein was identified as hnRNPL. **(a)** The most discriminative peak (m/z signals) in MS. **(b-l)** The most discriminative peaks (m/z signals) in MS/MS.

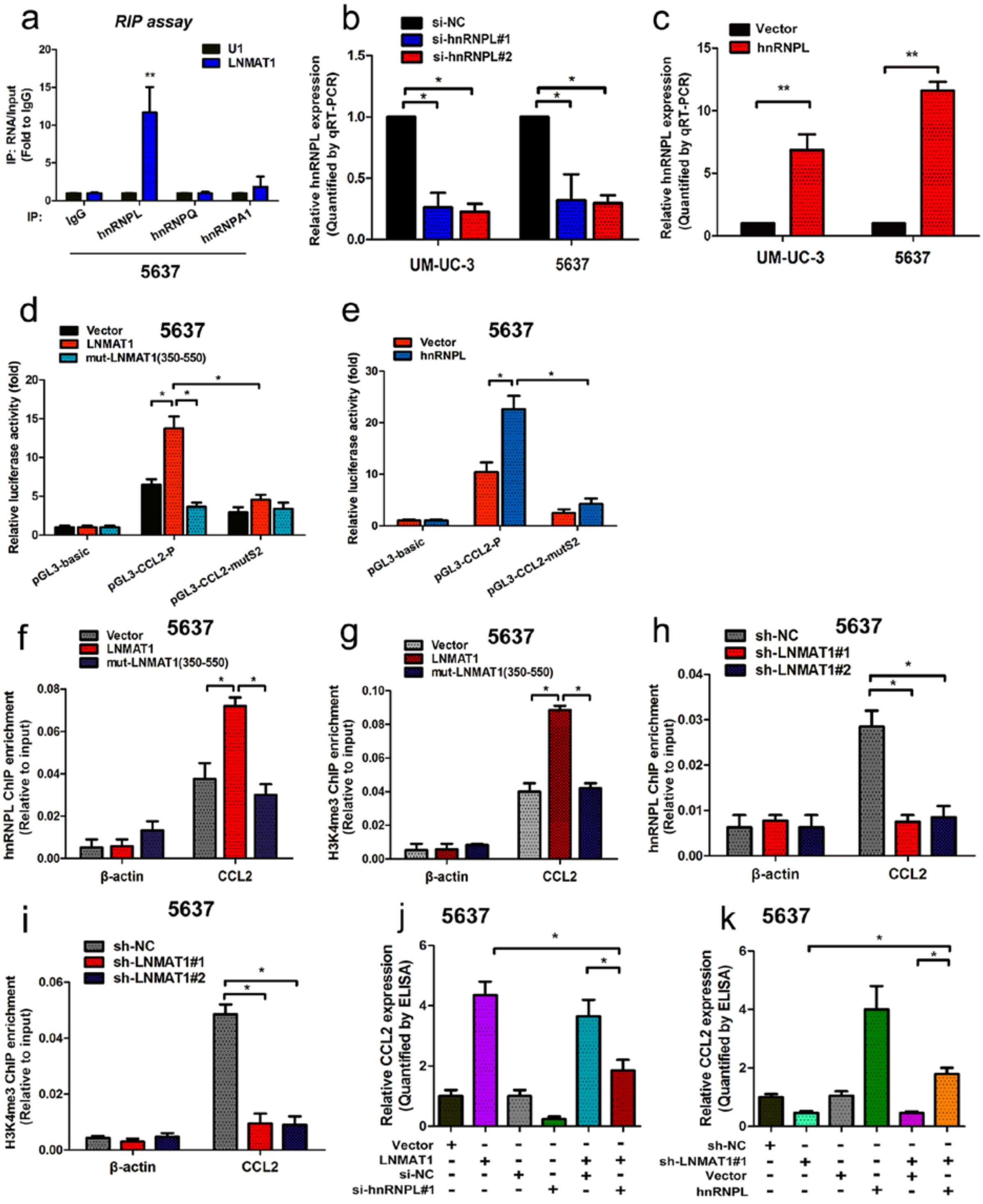

Supplementary Figure 9.｜*LNMAT1* transcriptionally upregulates CCL2 expression. (a) RIP analysis using the anti-hnRNPL antibody revealed that *LNMAT1* interacted with endogenous hnRNPL in 5637 cells. Statistical significance was assessed using two-tailed t-tests. **(b,c)** qRT-PCR analysis of hnRNPL expression in hnRNPL-transduced, hnRNPL-silenced and control cell as indicated. **(d,e)** CCL2 wild-type (-2000 ~ +121) or

LNMAT1 binding site mutated promoter (CCL2-mutS2) were constructed in the pGL3 vector and subjected to luciferase reporter assays in *LNMAT1* or hnRNPL overexpressing cells. Statistical significance was assessed using one-way analyses of variance (ANOVA) followed by Dunnett's tests and two-tailed t-tests. **(f-i)** ChIP-qPCR analysis of hnRNPL occupancy and H3K4me3 status in the CCL2 promoter after overexpression/knock down of *LNMAT1* in 5637 cells as indicated. Statistical significance was assessed using one-way analyses of variance (ANOVA) followed by Dunnett's tests and two-tailed t-tests. **(j,k)** ELISA analyses of CCL2 expression in the hnRNPL overexpression or knockdown on *LNMAT1*-induced CCL2 expression in 5637 cells. Statistical significance was assessed using one-way analyses of variance (ANOVA) followed by Dunnett's tests and two-tailed t-tests.The error bars represent standard deviations of three independent experiments. *$p < 0.05$ and **$p < 0.01$.

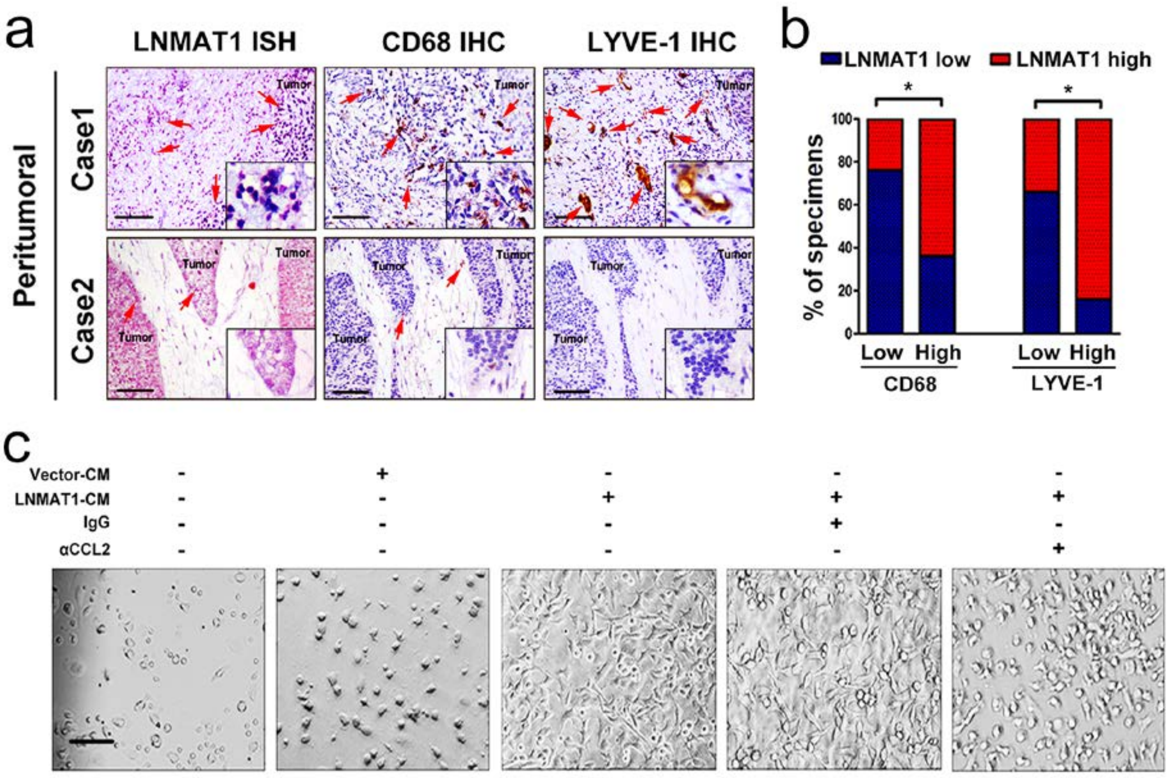

Supplementary Figure 10. | ***LNMAT1*-upregulated CCL2 activates TAMs. (a,b)** Representative images and percentages of bladder cancer tissues with high or low levels of CD68-positive cells and LYVE-1-positive cells in the peritumoral tissues with different *LNMAT1* expression levels. *LNMAT1* expression levels were quantified by ISH, macrophage density was quantified by IHC using anti-CD68 antibody and microlymphatic vessel density was quantified by IHC using the anti-LYVE-1 antibody. Two representative cases are shown. Statistical significance was assessed by χ^2 test. Scale bars: 100 μm. **(c)** Representative images of macrophages treated with conditioned medium collected from the indicated cells. Scale bars: 100 μm. The error bars represent standard deviations of three independent experiments. *$p < 0.05$, and **$p < 0.01$.

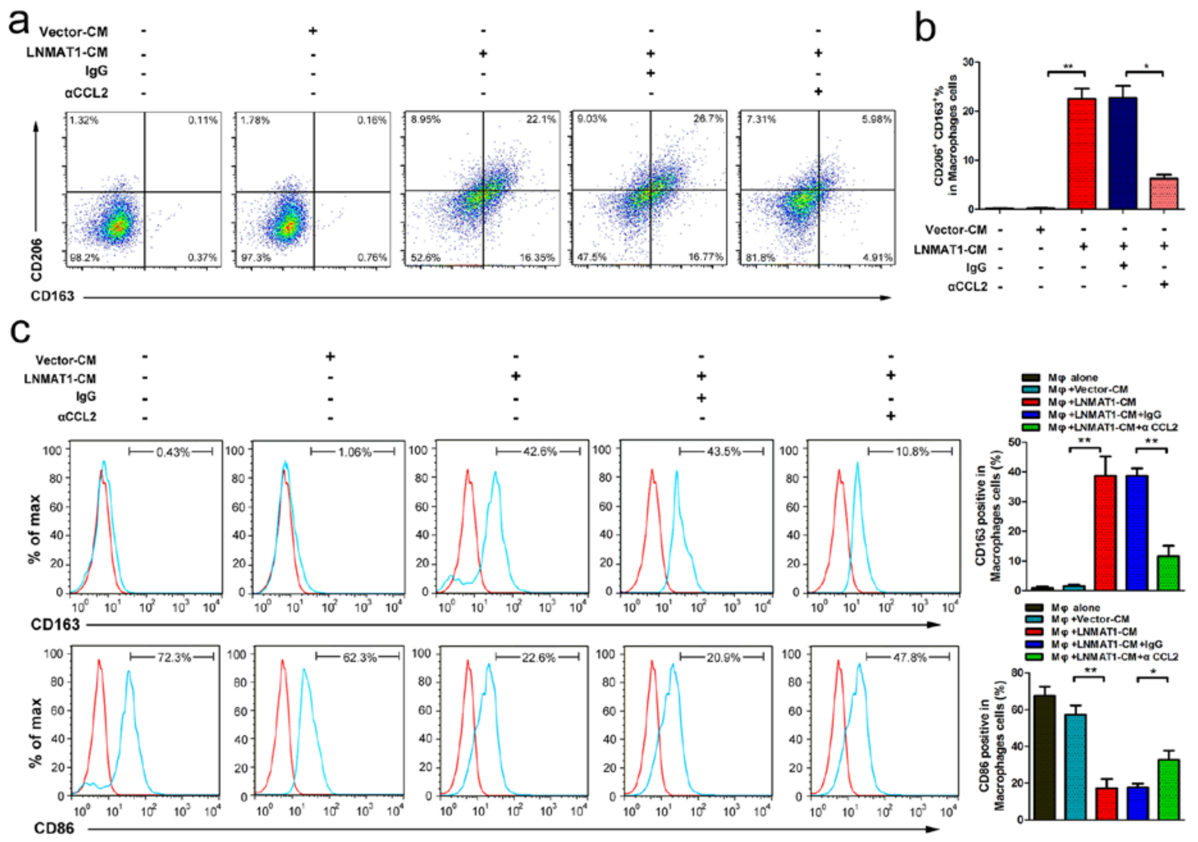

Supplementary Figure 11. | ***LNMAT1*-upregulated CCL2 induces TAMs recruitment.** **(a,b)** Flow cytometric analysis (a) and quantification (b) of the expressions of the TAMs marker CD206$^+$ and CD163$^+$ in macrophages treated with medium collected from the indicated cells for 24 hr. CCL2-neutralizing treatment was carried out for an additional 18 hr. Statistical significance was assessed using two-tailed t-tests. **(c)** Flow cytometric analysis and quantification of the expressions of CD163/CD86 in macrophages treated with medium collected from the indicated cells for 24 hr. CCL2-neutralizing treatment was carried out for an additional 18 hr. Statistical significance was assessed using two-tailed t-tests. The error bars represent standard deviations of three independent experiments. *$p < 0.05$, and **$p < 0.01$.

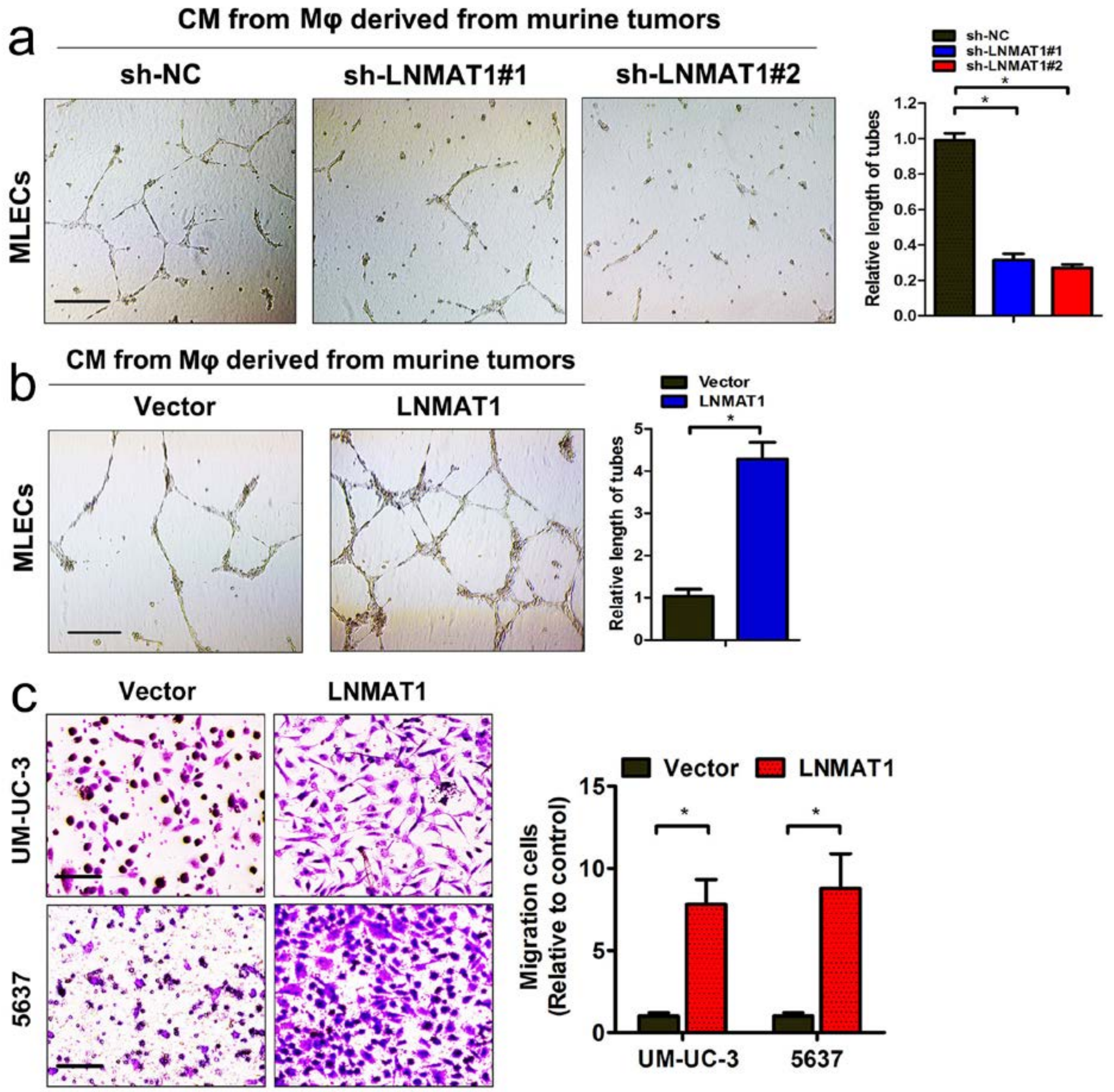

Supplementary Figure 12.｜CCL2-activated macrophages induce lymphangiogenesis. (a,b) Representative images (left panel) and quantifications (right panel) of tube formation by MLECs treated with CM derived from TAMs sorted from the indicated murine tumors. Statistical significance was assessed using one-way analyses of variance (ANOVA) followed by Dunnett's tests for multiple comparison and two-tailed t-tests. **(c)** *LNMAT1* overexpression promoted the migratory capability of bladder cancer cells through lymphatic endothelium monolayer. Scale bars: 100 µm. Statistical significance was assessed using two-tailed t-tests. The error bars represent standard deviations of three independent experiments. *$p < 0.05$, and **$p < 0.01$.

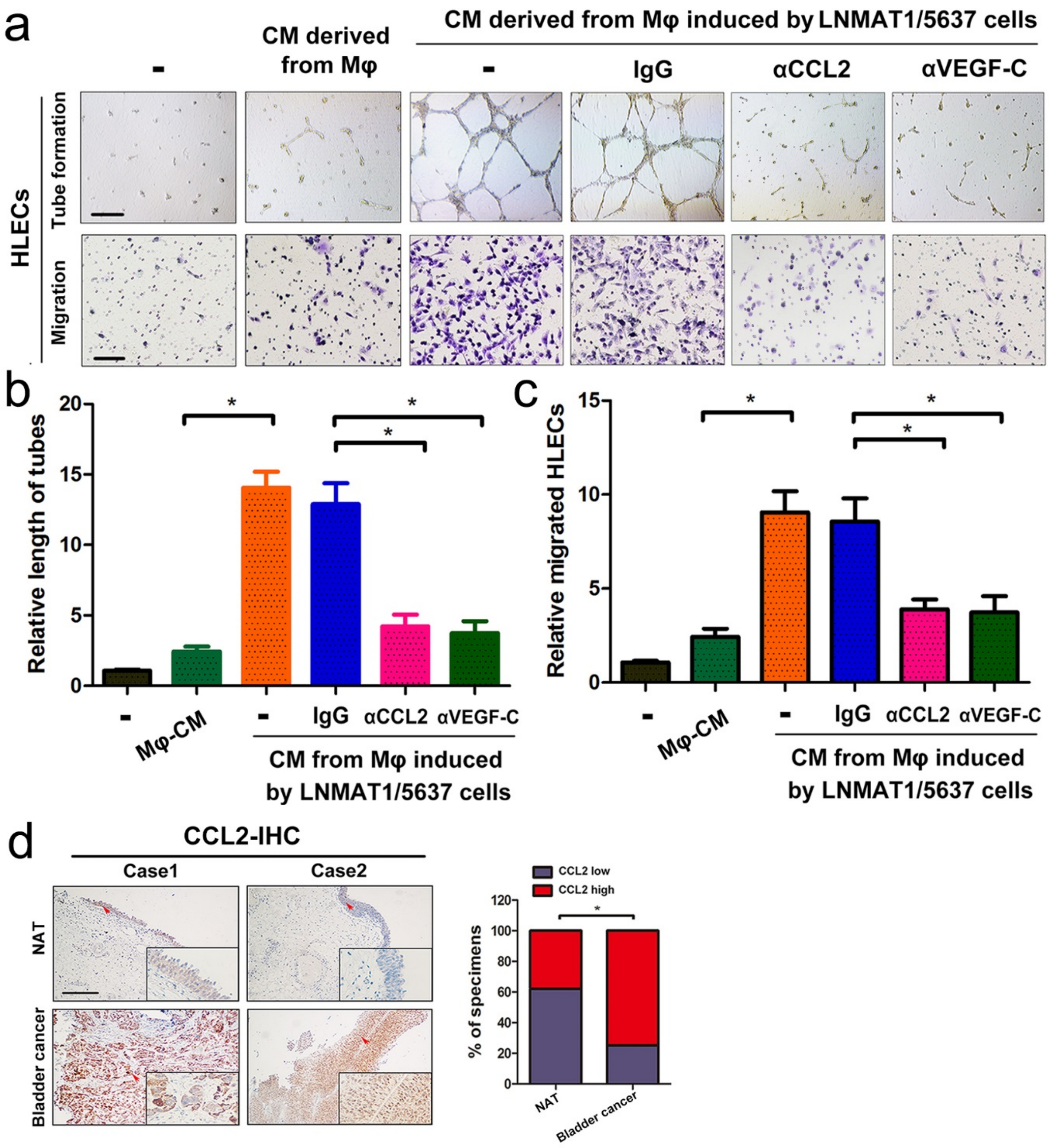

Supplementary Figure 13. | ***LNMAT1*****-upregulated CCL2 induces LN metastasis of bladder cancer. (a-c)** Representative images (a) and quantifications (b,c) of tube formation and Transwell migration by HLECs treated with conditioned medium (CM) collected from macrophages induced by *LNMAT1* from 5637 cells. HLECs were cultured with CM derived from TAMs induced by the indicated cancer cells. Scale bars: 100 µm. Statistical significance was assessed using one-way analyses of variance (ANOVA) followed by Dunnett’s tests for multiple comparison and two-tailed t-tests. **(d)** IHC staining shows that CCL2 expression is upregulated in human bladder cancer tissues compared with NAT (n = 266). Scale bar: 100

μm. Statistical significance was assessed by χ^2 test. The error bars represent standard deviations of three independent experiments. *$p < 0.05$, and **$p < 0.01$.

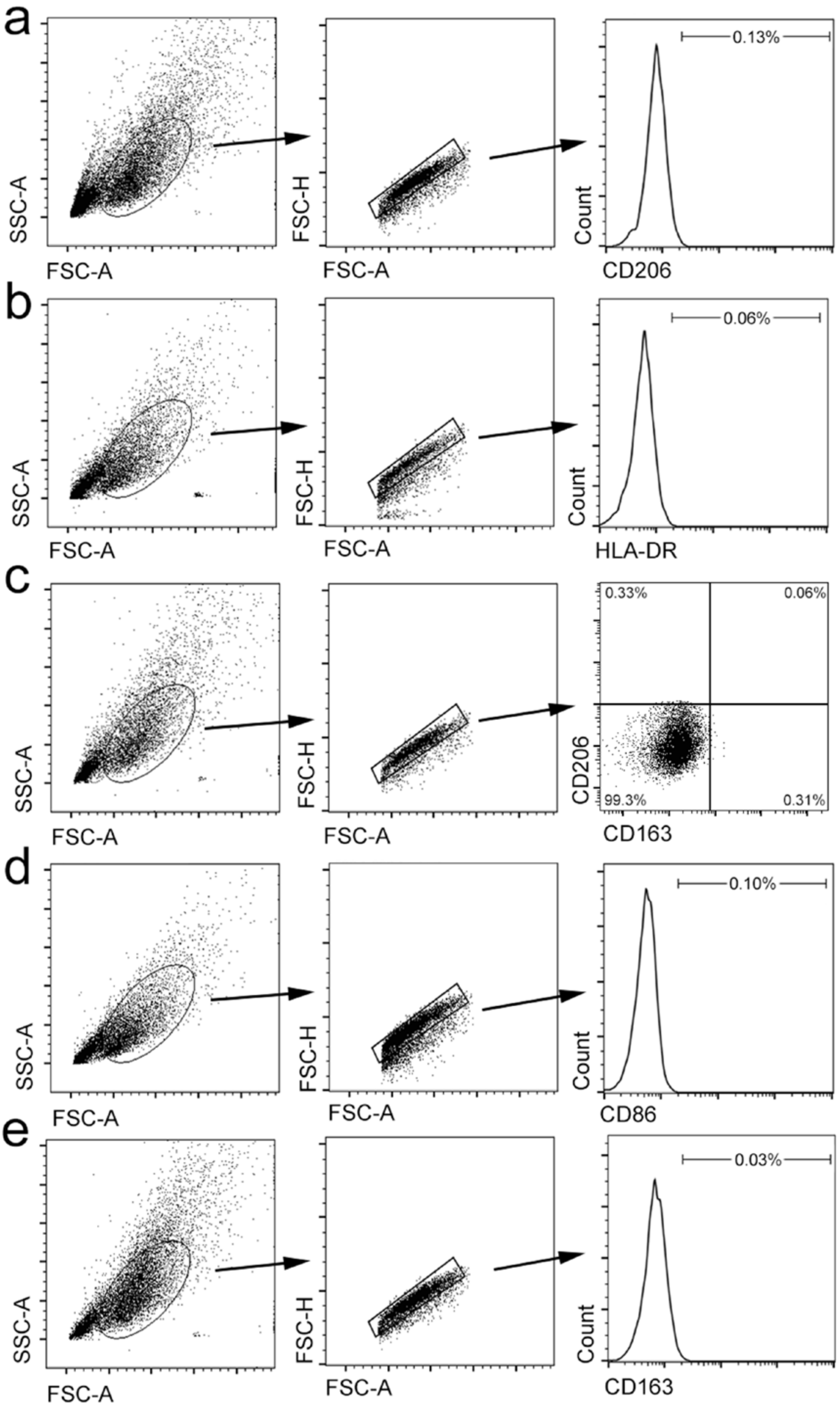
a
SSC-A
FSC-A
FSC-H
FSC-A
Count
CD206
0.13%
b
SSC-A
FSC-A
FSC-H
FSC-A
Count
HLA-DR
0.06%
c
SSC-A
FSC-A
FSC-H
FSC-A
CD206
CD163
0.33%
0.06%
99.3%
0.31%
d
SSC-A
FSC-A
FSC-H
FSC-A
Count
CD86
0.10%
e
SSC-A
FSC-A
FSC-H
FSC-A
Count
CD163
0.03%

Supplementary Figure 14.｜Gating strategies used for cell surface markers analysis. (a) Gating strategy to analyze CD206 positive cells from macrophages treated with medium collected from the indicated cells for 24 hr presented on Fig. 7c. **(b)** Gating strategy to analyze HLA-DR positive cells from macrophages treated with medium collected from the indicated cells for 24 hr presented on Fig. 7c. **(c)** Gating strategy to analyze CD163/CD206 positive cells from macrophages treated with medium collected from the indicated cells for 24 hr presented on Supplementary Fig. 11a,b. **(d)** Gating strategy to analyze CD86 positive cells from macrophages treated with medium collected from the indicated cells for 24 hr presented on Supplementary Fig. 11c. **(e)** Gating strategy to analyze CD163 positive cells from macrophages treated with medium collected from the indicated cells for 24 hr presented on Supplementary Fig. 11c.

Full uncut original picture for Figure 2F

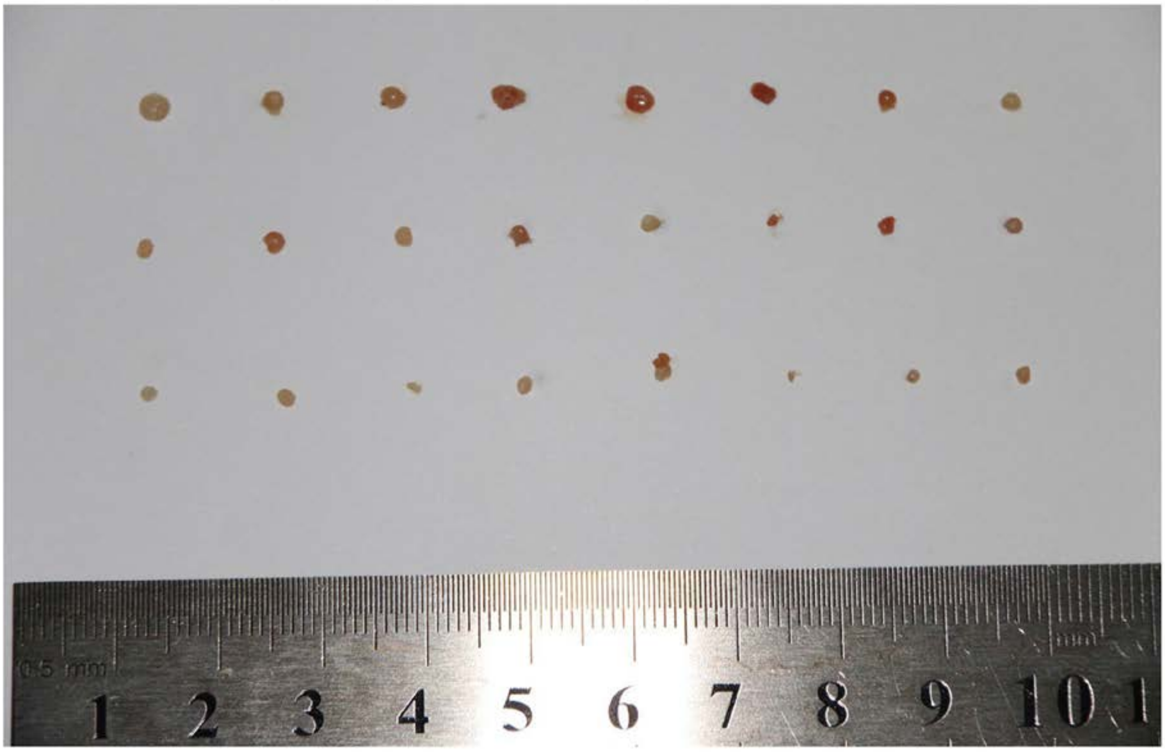

Full uncut original picture for Supplementary Figure 2C

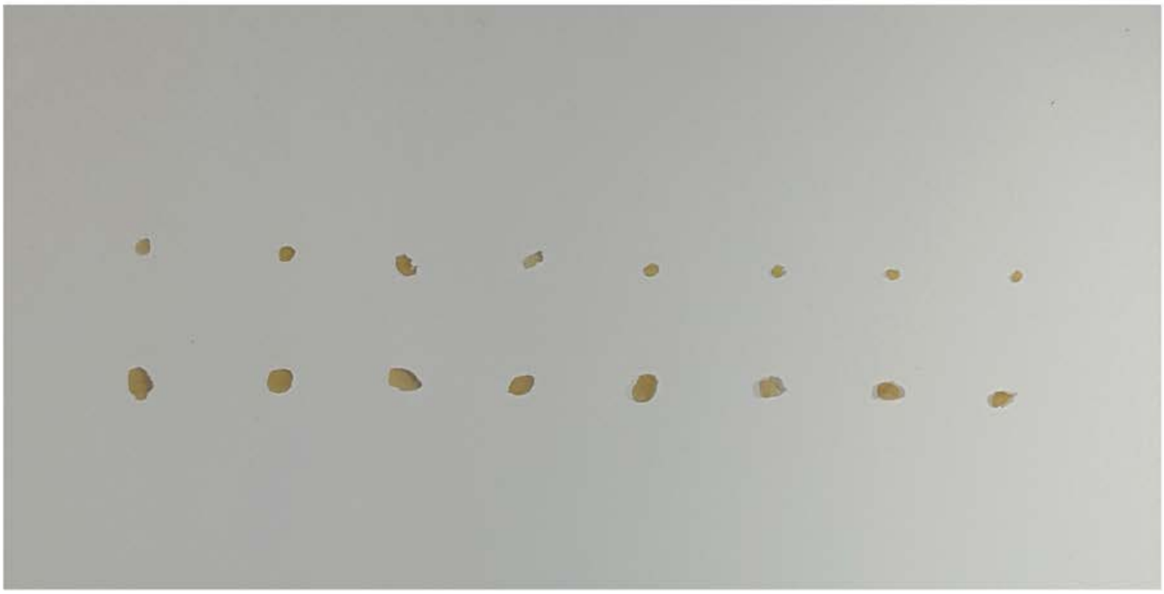

Supplementary Figure 15.｜Full uncut original pictures *in vivo*.

Full uncut original pictures

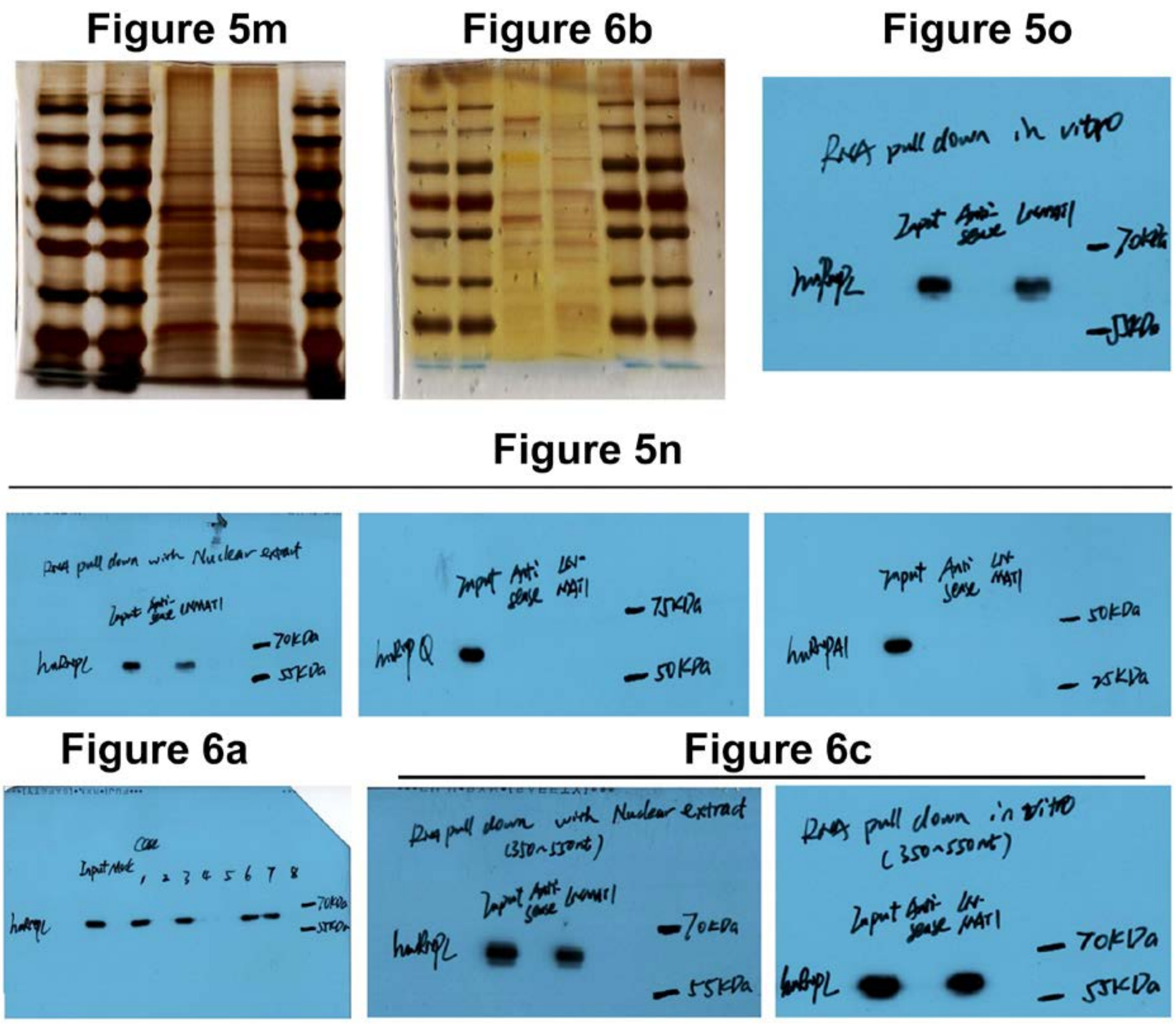

Supplementary Figure 16. | Full uncut original pictures.

Supplementary Table 1. Patient characteristics for next-generation sequencing (NGS).

Patient	Gender	Age	TNM stage	Pathological grade
BCa1/NAT1	Male	61	T3bN0M0	Ⅲ
BCa2/NAT2	Male	58	T2aN1M0	Ⅳ
BCa3/NAT3	Female	72	T3aN1M1	Ⅳ
BCa4/NAT4	Female	68	T3bN0M0	Ⅲ
BCa5/NAT5	Male	74	T2bN1M0	Ⅳ
LN(+)	Female	68	T3bN1M0	Ⅳ
LN(+)	Male	72	T3aN1M0	Ⅳ
LN(+)	Male	52	T3aN2M0	Ⅳ
LN(+)	Male	76	T3bN1M0	Ⅳ
LN(+)	Male	71	T3aN1M0	Ⅳ
LN(-)	Female	65	T3bN0M0	Ⅲ
LN(-)	Male	76	T3aN0M0	Ⅲ
LN(-)	Male	51	T3aN0M0	Ⅲ
LN(-)	Male	76	T3bN0M0	Ⅲ
LN(-)	Male	68	T3aN0M0	Ⅲ

Abbreviation：BCa = bladder cancer; NAT = normal adjacent tissue; T stage = tumor stage; T grade = tumor grade; LN = lymph node.

Supplementary Table 2. Correlation between *LNMAT1* and CCL2 expression and clinicopathologic characteristics of bladder cancer patients.

Characteristics	Patient frequency (%)	*LNMAT1* expression level			CCL2 expression level		
		Low	High	*p*-value[a]	Low	High	*p*-value[a]
**Gender**				0.567			0.227
Female	65	32	33		36	29	
Male	201	89	112		94	107	
**Age**				0.533			0.353
<65	112	48	64		51	61	
≥65	154	73	81		79	75	
**T stage**				0.185			0.908
<T2	83	43	40		41	42	
≥T2	183	78	105		89	94	
**T grade**				**0.008****			0.846
Low	71	42	29		34	37	
High	195	79	116		96	99	
**LN metastasis**				**0.004****			**0.003****
LN(-)	209	105	104		112	97	
LN(+)	57	16	41		18	39	

Abbreviation：T stage = tumor stage; T grade = tumor grade; LN = lymph node; [a] Chi-square test, *$p < 0.05$, **$p < 0.01$.

Supplementary Table 3. Univariate and multivariate Cox regression analyses of overall survival (OS) in bladder cancer patients.

`	Univariate analysis			Multivariate analysis		
	**HR**	**95% CI**	***p*-Value**	**HR**	**95% CI**	***p*-Value**
**Age (>65 vs ≤65)**	0.715	0.4755-1.075	0.107			
**Gender (Male vs Female)**	1.133	0.703-1.827	0.609			
**T stage (≥T2 vs ＜T2)**	1.188	0.755-1.867	0.456			
**T grade (High vs Low)**	1.758	1.085-2.850	**0.022***	1.712	1.053-2.782	**0.030***
**LN metastasis (LN^+ vs LN^-)**	1.967	1.232-3.143	**0.005****	1.715	1.063-2.768	**0.027***
***LNMAT1* expression (High vs Low)**	1.828	1.189-2.810	**0.006****	1.638	1.055-2.544	**0.028***

Abbreviations: HR = hazard ratio; 95% CI = 95% confidence interval; T stage = tumor stage; T grade = tumor grade; LN = lymph node; *$p < 0.05$, **$p < 0.01$.

Supplementary Table 4. Univariate and multivariate Cox regression analyses of disease free survival (DFS) in bladder cancer patients.

`	Univariate analysis			Multivariate analysis		
	**HR**	**95% CI**	***p*-Value**	**HR**	**95% CI**	***p*-Value**
**Age (>65 vs ≤65)**	0.796	0.541-1.094	0.144			
**Gender (Male vs Female)**	0.921	0.622-1.364	0.682			
**T stage (≥T2 vs ＜T2)**	1.164	0.791-1.712	0.441			
**T grade (High vs Low)**	1.741	1.156-2.624	**0.008****			
**LN metastasis (LN^+ vs LN^-)**	1.923	1.276-2.898	**0.002****	1.650	1.086-2.508	**0.019***
***LNMAT1* expression (High vs Low)**	1.903	1.317-2.750	**0.001****	1.724	1.183-2.511	**0.005****

Abbreviations: HR = hazard ratio; 95% CI = 95% confidence interval; T stage = tumor stage; T grade = tumor grade; LN = lymph node; **p* < 0.05, ***p* < 0.01.

Supplementary Table 5. The possible TFO predicted by LongTarget for *LNMAT1* and CCL2 promoter.

Oligo ID	TFO (5'-3')	Oligo ID	TTS (5'-3')	Strand	Score
TFO1	CTCTGCTTCCTGGGTTTAC	TTS1	GAGAGAGGACCCAAGCAG	AntiPlus	42
TFO2	CTCCACCGTCGCCACA	TTS2	GAGGTGGCAGCCCACT	ParaMinus	34
TFO3	ATTCTCCTGCCTCAGCC	TTS3	TAAGAGGCAGAGACAG	AntiPlus	41
TFO4	AGGCTGGAGTGCAGTGG	TTS4	TCCGCCCTCTCTCCCTC	AntiMinus	71
TFO5	TCCCTTCATCTGTGGATG	TTS5	ATGGAAGATCCCTCCTCC	ParaPlus	62
TFO6	AAAGGATGAAATTTCATT	TTS6	TTTCCTACTTCCTGGAAA	AntiPlus	52

Abbreviation：TFO = Triplex-forming oligos; TTS = Triplex target sites; Score = Triplex-forming potential score.

Supplementary Table 6. Primer of experiments.

Primer name	Forward primer (5'-3')	Reverse primer (5'-3')	Application
LNMAT1	CTGGGTTCACGCCATTCT	CCCATTCTCGTATGGAGGTT	qRT-PCR
LNMAT1 Primer A (350-550nt)	CAGTATGGCTCCAAAGGATG	CGAGACTGCCCATCCACA	qRT-PCR
LNMAT1 Primer B	AGTCACTCACATCCCTCCAC	CGAGTGTGACAGTGAAGTGC	qRT-PCR
GAPDH	TGGTGAAGACGCCAGTGGA	GCACCGTCAAGGCTGAGAAC	qRT-PCR
U1	GGGAGATACCATGATCACGAAGGT	CCACAAATTATGCAGTCGAGTTTCCC	qRT-PCR
hnRNPL	TGTAATCCTTGTGGCCCTGT	ATCAGCCCCATTGAGAGAGG	qRT-PCR
CCL2	TTCCCTGGCCTGAAGTTCTT	CTTGGTGCCTCAGTTTTCCC	qRT-PCR
VEGF-C	GGCTGGCAACATAACAGAGAA	CCCCACATCTATACACACCTCC	qRT-PCR
MALAT1	GACGGAGGTTGAGATGAAGC	ATTCGGGGCTCTGTAGTCCT	qRT-PCR
U6	CTCGCTTCGGCAGCACATATAC	AACGCTTCACGAATTTGCGTGTC	qRT-PCR
β-actin	GAAGCTAAGTCCTGCCCTCA	CAGTGAGGACCCTGGATGTG	qRT-PCR
CCL2-S1	AATCAATGCCCCAGTCACCT	TTGCTGCTGGTGATTCTTCT	ChIRP
CCL2-S2	GCTCAGCAGATTTAACAGCC	GCTGCTGTCTCTGCCTCTTA	ChIRP
CCL2-S3	TGCTGTCTATGCCTTTGTCC	AGCATCATAGAAGCCTAGCAGA	ChIRP
CCL2-S4	GGACAGAGAGAGGACCCAAG	GCTGCCACCTCTCTGACATA	ChIRP
CCL2-S5	ACATTGCCTCAGTGACCCTT	TCGATTCTCCTAGCCCACTG	ChIRP
GAPDH-RNA	CAAGGCTGAGAACGGGAAG	AGGTAGTTTCGTGGATGCCA	ChIRP
GAPDH-DNA	GTTTCCAGGAGTGCCTTTGTG	ATTAGGGCAGACAATCCCGGC	ChIRP
CCL2	GCTCAGCAGATTTAACAGCC	GCTGCTGTCTCTGCCTCTTA	ChIP-qPCR
β-actin	GAAGCTAAGTCCTGCCCTCA	CAGTGAGGACCCTGGATGTG	ChIP-qPCR
LNMAT1 5'RACE	Universal primer in SMARTer kit	GTGCCTGTAATCCCAGCTACTCA	Nested PCR (Outer)
LNMAT1 5'RACE	Universal primer in SMARTer kit	GAGGCAGTCTCAGAAGGAATCAGT	(Inner)
LNMAT1 3'RACE	CTCCTGAGTAGCTGGGATTACAGG	Universal primer in SMARTer kit	(Outer)

LNMAT1 3'RACE	CACACTCAGAGTCACTCACATCC	Universal primer in SMARTer kit	(Inner)
sh-LNMAT1#1	GGCUGGAGAAUAUUUCCUATT	UAGGAAAUAUUCUCCAGCCTT	shRNAs
sh-LNMAT1#2	GAGAAUGGGUCUAAAGGAATT	UUCCUUUAGACCCAUUCUCTT	shRNAs
sh-CCL2#1	CAAACCCAAACUCCGAAGATT	UCUUCGGAGUUUGGGUUUGTT	shRNAs
sh-CCL2#2	CUCGCGAGCUAUAGAAGAATT	UUCUUCUAUAGCUCGCGAGTT	shRNAs
si-hnRNPL#1	CACUGGUGGAGUUUGAAGATT	UCUUCAAACUCCACCAGUGTT	siRNAs
si-hnRNPL#2	CCCAUUUAUUCGAUCACCATT	UGGUGAUCGAAUAAAUGGGTT	siRNAs
si-CCL2#1	GCUGUUAUAACUUCACCAATT	UUGGUGAAGUUAUAACAGCTT	siRNAs
si-CCL2#2	CUCGCGAGCUAUAGAAGAATT	UUCUUCUAUAGCUCGCGAGTT	siRNAs

Supplementary Table 7. Probes used in In situ hybridization (ISH) and Chromatin isolation by RNA purification (ChIRP).

Probe name	Probe sequences (5’-3’)	Label	Application
LNMAT1	ATGGAGGTTTGTTTTCTTCTT	5’-DIG labeled and 3’-DIG labeled	ISH
U6	CACGAATTTGCGTGTCATCCTT	5’-DIG labeled and 3’-DIG labeled	ISH
Scramble	GTGTAACACGTCTATACGCCCA	5’-DIG labeled and 3’-DIG labeled	ISH
*LNMAT1*_odds1	TACTCCACCATCACATGGAA	3’-Biotin	ChIRP
*LNMAT1*_odds2	CTGCTGAGGAGCAAGACATC	3’-Biotin	ChIRP
*LNMAT1*_odds3	CATACCTTGCTCAACTACTC	3’-Biotin	ChIRP
*LNMAT1*_odds4	ACGCGAGTGTGACAGTGAAG	3’-Biotin	ChIRP
*LNMAT1*_even1	ATTCTCGTATGGAGGTTTGT	3’-Biotin	ChIRP
*LNMAT1*_even2	TATGGGAAGGGGACTGTCTG	3’-Biotin	ChIRP
*LNMAT1*_even3	TGTGAGTGACTCTGAGTGTG	3’-Biotin	ChIRP
*LNMAT1*_even4	CCTTTATTAAGACCTGTGGG	3’-Biotin	ChIRP

Abbreviation：Digoxigenin = DIG

Supplementary Methods

RNA-Seq and data analysis

Total RNA was extracted from bladder cancer and normal samples with TRIzol reagent. The RNA purity and integrity were analyzed with the Agilent Bioanalyzer 2100 (Agilent). Qualified total RNA was further purified with the RNeasy mini kit (QIAGEN) and RNase-free DNase set (QIAGEN). The mRNA was purified from total RNA after removal of rRNA and sequenced on an Illumina Hiseq2000 system.

RNA extraction, cDNA synthesis and quantitative real-time PCR (qRT-PCR) analysis

Total RNA was extracted using the TRIzol reagent (Life Technologies, Carlsbad, CA, USA) and treated with RNase-free DNase (QIAGEN, Germantown, MD, USA) following the manufacturers' instructions. The cDNA was synthesized using 500 ng of total RNA and with SuperScript II reverse transcriptase (Invitrogen, San Diego, CA, USA). qRT-PCR analysis was conducted using the SYBR Green master mix (Roche, CA, USA) and analyzed on a Roche Light-Cycler system (Roche, CA, USA). The relative expression levels were calculated using the $2^{-\Delta\Delta CT}$ method, with GAPDH as an internal control. All specific primers used in the study are listed in Supplementary Table 6.

5' and 3' Rapid amplification of cDNA ends (RACE) of LNMAT1

5' and 3' RACE assays were performed to determine the transcriptional initiation and termination sites of *LNMAT1* with a SMARTer RACE 5'/3' kit according to the manufacturer's instructions (Clontech Laboratories, Mountain View, CA, USA). The sequences for the *LNMAT1*-specific nested PCR primers used for 5' and 3' RACE analysis are provided in Supplementary Table 6.

RNA fluorescence in situ hybridization (FISH) and nuclear fractionation

A FISH kit (Ribo Bio, Guangzhou, China) was used according to the manufacturer's protocol, and fluorescence was visualized by confocal microscopy (Zeiss, Oberkochen, Germany). Bladder cancer cells were seeded, fixed with 4% paraformaldehyde, treated with 0.5% Triton in phosphate buffer saline (PBS), and prehybridized. The cells were hybridized with 5 μM CY3 probe overnight. The CY3-labeled U6, 18S, and the labeled FISH probes were provided by Ribo Bio (Guangzhou, China). The cellular fraction was isolated as described previously. Briefly, 1×10^7 cells were harvested, washed in 1 ml of ice-cold RNase-free PBS, washed in 1 ml of buffer C1 (1.28 M Sucrose, 40 mM Tris-HCl, pH 7.5, 20 mM $MgCl_2$, 4% Triton X-100), washed in 3 ml of RNase-free water, and incubated for 15 min on ice. Then, cells were centrifuged for 15 min at 2500 rpm, and the supernatant containing the cytoplasmic fraction was kept together with the pellet containing the nuclear fraction for RNA extraction.

Serial deletion analysis and site-directed mutagenesis.

The serial 3' nested PCR primers with common 5' primers or serial 5' nested PCR primers with common 3' primers were used to amplify the serial deletion fragments of *LNMAT1*. For in vitro transcription of biotin-labeled and unlabeled *LNMAT1* RNA, full-length *LNMAT1* or mutant *LNMAT1* RNAs, carrying various deletions, were cloned into the pcDNA3.1 vector for the RNA pull-down assays. The mutant *LNMAT1* RNAs (Δ350-550) described in the manuscript were generated using a QuikChange Site-directed Mutagenesis Kit (Stratagene, La Jolla, CA, USA) according to the manufacturer's instructions.

ELISA analysis

The cell culture supernatant was collected, secreted CCL2 was quantified using the Human CCL2/MCP-1 Quantikine ELISA Kit (Cat. No. DCP00, R&D), and secreted VEGF-C was quantified using the Human VEGF-C Quantikine ELISA Kit (Cat. No. DVEC00, R&D)

according to the manufacturer's instructions.

Lentivirus-mediated transduction

The full-length *LNMAT1* was amplified by PCR, cloned into the pCDH-CMV-MCS-EF1-Puro (with or without luciferase) or pLKO.1-Puro (with or without luciferase) vector and bidirectionally sequenced. After 72 h of transfection, the viral supernatants of 293T cells were collected. Lentiviral particles were concentrated using a LentiX™ Concentrator overnight at 4°C. UM-UC-3 and 5637 cells (5×10^5 cells per well) were seeded in six-well culture plates and infected with lentivirus and polybrene for 24 hr. Cells were infected with lentivirus in the presence of polybrene (Sigma-Aldrich) and selected with puromycin (Sigma-Aldrich) for 2 weeks to obtain stable expression cell lines. The shRNA sequences are provided in Supplementary Table 6.

Cell transfection and luciferase activity assay

The UM-UC-3 and 5637 cells were transfected with small interfering RNA (siRNA) or plasmids using Lipofectamine RNAi Max (Life Technologies, CA, USA) according to the manufacturer's instructions. All siRNAs were purchased from GenePharma (Shanghai, China). After 48 hr, the efficiency of siRNA knockdown was assessed by qRT-PCR. Bladder cancer cells were lysed, and luciferase activity was measured using the Dual Luciferase Assay System (Promega) and the SpectraMax M5 reader (Molecular Devices, CA, USA). All transfections were carried out in triplicate following the manufacturer's instructions. The siRNA sequences are provided in Supplementary Table 6.

Wound healing assay

The cells were seeded at 4×10^4 cells per cm^2 and were grown to 90% confluency. The cell layer was scrapping with a 10-μl sterile pipette tip and washed with three times with PBS to remove the floating and detached cells. Fresh serum-free medium was added, and images

were acquired using a microscope at different time points (0, 12, 24 and 48 hr).

MLECs tube formation assay

The MLECs were isolated from C57BL/6 mice (approximate age 2 months). MLECs were then seeded into 12-well plates (pre-coated with Matrigel) containing concentrated media and incubated for 12 hr. The resulting lymphatic tubes were photographed using an inverted microscope and quantified by measuring the number and area of the completed tubule structures.

Flow cytometry assay

Cell surface marker analysis of monocytes/macrophages was performed by flow cytometry. A total of 1×10^6 cells was resuspended in PBS containing 2% FBS in each test tube and kept on ice for 10 min. Corresponding antibodies (CD206, ab64693 and CD68, CST #76437) and the appropriate secondary antibodies were added to the cell suspensions, and the mixtures were incubated on ice in the dark for 45 min. The cells were washed and resuspended in 500 ml of FACS buffer and then analyzed using FlowJo software (Tree Star, Ashland, OR, USA).
